# Self-Assembled CNF/rGO/Tannin Composite: Study of the Physicochemical and Wound Healing Properties

**DOI:** 10.3390/polym15122752

**Published:** 2023-06-20

**Authors:** Katherina Fernández, Aylen Llanquileo, Monserrat Bustos, Valentina Aedo, Isleidy Ruiz, Sebastián Carrasco, Mauricio Tapia, Miguel Pereira, Manuel F. Meléndrez, Claudio Aguayo, Leonard I. Atanase

**Affiliations:** 1 Laboratorio de Biomateriales, Departamento de Ingeniería Química, Facultad de Ingeniería, Universidad de Concepción, Concepción 4070386, Chile; ayllanquileo@udec.cl (A.L.); monserratbustos@udec.cl (M.B.); vaedo2017@udec.cl (V.A.); isruiz@udec.cl (I.R.); scarrasco2017@udec.cl (S.C.); mtapia2017@udec.cl (M.T.); 2 Laboratorio de Productos Forestales, Departamento de Ingeniería Química, Facultad de Ingeniería, Universidad de Concepción, Concepción 4070386, Chile; miguelpereira@udec.cl; 3 Grupo Interdisciplinario de Nanotecnología Aplicada (GINA), Laboratorio de Materiales Híbridos (HML), Departamento de Ingeniería de Materiales (DIMAT), Facultad de Ingeniería, Universidad de Concepción, Concepción 4070386, Chile; mmelendrez@udec.cl; 4 Departmento de Inmunología y Bioquímica Clínica, Facultad de Farmacia, Universidad de Concepción, Concepción 4070386, Chile; caguayo@udec.cl; 5 Faculty of Medical Dentistry, Apollonia University of Iasi, 700511 Iasi, Romania; 6 Academy of Romanian Scientists, 050045 Bucharest, Romania

**Keywords:** graphene oxide, nanocellulose, reduced graphene oxide, polydopamine, wound healing

## Abstract

In this study, a conductive composite material, based on graphene oxide (GO), nanocellulose (CNF), and tannins (TA) from pine bark, reduced using polydopamine (PDA), was developed for wound dressing. The amount of CNF and TA was varied in the composite material, and a complete characterization including SEM, FTIR, XRD, XPS, and TGA was performed. Additionally, the conductivity, mechanical properties, cytotoxicity, and in vitro wound healing of the materials were evaluated. A successful physical interaction between CNF, TA, and GO was achieved. Increasing CNF amount in the composite reduced the thermal properties, surface charge, and conductivity, but its strength, cytotoxicity, and wound healing performance were improved. The TA incorporation slightly reduced the cell viability and migration, which may be associated with the doses used and the extract’s chemical composition. However, the in-vitro-obtained results demonstrated that these composite materials can be suitable for wound healing.

## 1. Introduction

Skin is the largest body organ and comprises approximately one-sixth of the body mass with the primary role of protecting the inner organs from the external environment [1]. Extreme loss of skin function and structure due to injury and illness will result in substantial physiological imbalance and may ultimately lead to major disability or even death [2]. To remediate diseased or damaged tissue, surgical intervention can be accomplished by anatomically localizing the wound borders, thereby closing the wound, and reducing infection or contamination from the external environment [3]. For wounds that are not large-surface or deep, a combination of new strategies, using advanced biomaterials science, cells, and biochemical cues (proteins/growth factors (GFs)), are currently available [4].

In particular, with the development of advanced biomaterials, many forms of dressings, such as nanofibers, membranes, foams, hydrocolloids, and hydrogels have been created [5]. Ideally, wound dressing should promote healing and prevent contamination. To accelerate healing, the dressings should provide a moist wound environment, remove excess wound exudates, provide thermal insulation, allow gaseous exchange, be impermeable to microorganisms, allow current circulation, and have low adherence to the wound site [6].

A wound electrical signal at the wound site regulates cell behaviors, promoting wound healing [7]. Previous studies have reported 4 μA/cm^2^ wound current measurement immediately after trauma, which gradually increased to 10 μA/cm^2^ after 60 min. These wound potentials can promote wound regeneration by inducing cell behaviors through electrical stimulation [8]. Unfortunately, most wound dressings are non-electroactive and are therefore unable to respond to physiological electrical signals at wound sites during healing.

Nevertheless, the incorporation of electroactive materials such as reduced graphene oxide (rGO) into the wound dressing could be accelerated, distributing endogenous electrical stimulation more effectively to tissues in the wound [9]. Carbon materials could have difficult to disperse in the matrix, restricting their functionality and mechanical properties, and present also some lack cell affinity [10]. Both problems can be overcome using a biocompatible matrix that supports the electroactive material, such as cellulose nanofibers (CNF).

CNFs are made from wood-derived fiber (pulp) that has been micro-refined to the nano level. One of the most important characteristics of nanocellulose is that it can be colloidally stable in an aqueous solution for a wide range of salt concentrations and pH [11]. Moreover, it can assemble with other nanoparticles in a colloidal suspension in order to be transformed into a gel that is utilized to create multifunctional composites [12]. Nevertheless, CNF are poor electrochemically and unstable, which can be overcome by adding rGO to develop CNF-rGO composites. The interaction in the material is formed through a hydrogen bond between the unreacted oxygen-containing groups existing on rGO surface and CNF hydrophilic polymer matrix, endowing the composite with superior properties through a conjugate structure [13].

rGO has a multipotential to produce advanced materials for tissue engineering that require electrical signal transmission to strengthen cell-cell interactions [14]. rGO is produced from graphene oxide (GO), a graphene derivative carrying a functional group [12], which can interact with CNF and simultaneously be partially reduced by dopamine (DA) [15]. DA is generally considered to be a type of cell-compatible molecule, which can be strongly adsorbed into a majority of substrate materials through covalent conjugation or physical interactions [16]. In a weak alkaline pH, DA will simultaneously undergo self-polymerization to generate a hydrophilic polydopamine (PDA) coating on the surfaces of rGO to enhance its water dispersity [15]. Simultaneously, after the self-polymerization, PDA can be anchored on the GO nanosheets through Schiff or Michael’s addition reaction, and GO can be partially reduced [15]. Thus, the obtained rGO not only presents good dispersity and good electrical conductivity but also possesses excellent photothermal, antibacterial properties and bioactivity from PDA, which contribute to producing a bioactive wound dressing.

Condensed tannins (TA) are phenolic compounds, composed of flavan-3-ols, obtained from natural sources as vegetal and fruits [17]. They elicit several bioactivities [18] that can be transferred to a dressing, such as antioxidant, antimicrobial, and anti-inflammatory activities, among others, which could be favorable for the wound healing [19,20]. TA also reduce GO similarly to DA [21]. Phenolic groups in TA also aid anchoring to the rGO surface through π-π interactions [22]. It is expected with the TA incorporation into the composite material, the mechanical properties can be improved, and they can diffuse from the matrix helping in the cicatrization.

**Figure 1:**
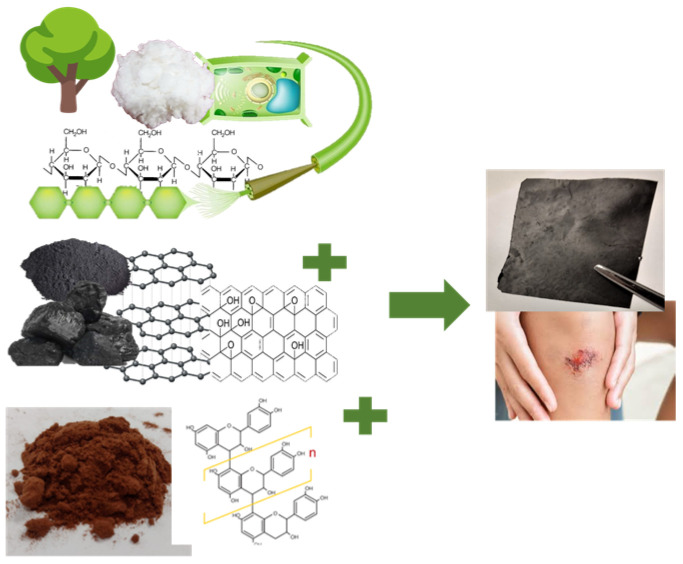
Schematic diagram of composite material synthesis.

Thus, this study developed CNF/rGO composites loaded with TA from pine bark, reduced with PDA, to be used as a wound dressing (Figure 1). To this purpose, the effect of different CNF concentrations (5, 15, 25, 50% *w*/*w*) on the CNF/rGO was evaluated and the materials were analyzed in terms of surface morphology, spectroscopy characterization, electric conductivity, swelling, traction and deformation, cell biocompatibility, and in vitro wound healing test.

## 2. Materials and Methods

### 2.1. Production of Cellulose Nanofibrils (CNF)

The bleached Kraft hardwood pulp (80% *Eucalyptus globulus* and 20% *Eucalyptus nitens*) used to produce CNFs was provided by the company CMPC pulp S.A. (Santa Fe Mill, Chile). The pulp was rehydrated, filtered, and pelleted according to ISO 5263. The chemical characterization of cellulosic fibers was performed through acid hydrolysis based on the method described by Zeng et al. [23] and Andrade et al. [24].

The enzymatic hydrolysis was performed using the commercial enzyme complex of cellulases Quimizime B (CHT Group, Santiago, Chile), which had a higher content of endoglucanases and an activity of 7.75 U/mL enzyme. Enzyme activity was determined according to the methodology of Ghose [25].

### 2.2. Mechanical-Enzymatic Pretreatment

Thirty grams of the oven-dried pulp of Kraft bleached eucalyptus pulp (BHKP) at 10% (*w*/*w*) were refined in a PFI (Hamjern Maskin A/S, Hamar, Norway) mill at 4000 revolutions to increase the accessibility of the enzyme in the substrate. Then, 0.05% of Quimizime B (CHT Group, Santiago, Chile) enzyme was added to the dry weight of the pulp. The enzymatic pretreatment conditions were: 60 min of reaction, temperature of 48 °C, 5% consistency, pH 5 (adjusted with 0.1 M HCl), and constant stirring at 800 rpm with a Stirrer Type BS (Velp Scientifica Srl, Usmate Velate, Italy). After the reaction time, the enzyme was denatured at 80 °C for 20 min. Next, the pulp was refined at a pressure of 700 bar using Gea Niro Soavi Panda Plus 2000 homogenizing equipment (Dusseldorf, Germany) provided with an S-type impact head. The fiber suspension was passed 15 times through the equipment to produce the CNFs; the final consistency was between 0.5–1%.

### 2.3. Synthesis of Graphene Oxide (GO)

GO was synthesized from natural graphite powder using the modified Hummers method [26]. Briefly, H_2_SO_4_ (Merck, Santiago, Chile) (270 mL) and H_3_PO_4_ (Merck, Santiago, Chile) (30 mL) were mixed in a beaker under stirring in an ice bath. Next, 2.25 g of graphite powder (Asbury, DeQuincy, LA, USA) and 13.5 g of KMnO_4_ (Merck, Santiago, Chile) were slowly added. The temperature was controlled not to exceed 45 °C, and the mixture was stirred for 1 h. The reaction was then stopped with H_2_O_2_ (Furet, Chillán, Chile) (60 mL) until a color change from brown to dark green was observed. The final mixture was centrifuged (Rotina 380R; Hettich, Germany) for 20 min at 5000 rpm, and the supernatant was removed. Subsequently, the oxidation product was continuously washed and centrifuged with ethanol (100 mL) and HCl (Merck, Santiago, Chile) (20 mL) to remove the metal ions (using AgNO_3_ (Merck, Santiago, Chile) as an indicator). The mixture was then washed four times with Milli-Q® water, and the supernatant was removed in each wash. The final mixture had a pH value of around 4. Next, the product was dialyzed in dialysis membranes (12000 MWCO) (Sigma-aldrich, Santiago, Chile) for three days to remove any remaining impurities and lyophilized for 72 h (Labconco freeze-dry system; Berlin, Germany) to obtain a solid dispersion of graphite oxide (GpO). Finally, the GpO was mechanically exfoliated with an Ultrasonic bath (Elmasonic E60H, Elma Schmidbauer GmbH, Singen am Hohentwiel, Germany) for 30 min to obtain a graphene oxide suspension. The samples were frozen in a cryogenic bath (−40 °C), lyophilizate to −40 °C and 1 × 10^−3^ mbar, and kept in a sealed flask for further use.

### 2.4. Pinus Radiata Bark Extract Production

*Pinus radiata* bark extracts were produced through a pilot-scale extraction process, as described by Bocalandro et al. [27]. For this purpose, a reactor volume of 4 m^3^ and a vapor heating system composed of a shell and a tube heat exchanger with 6 m^2^ heat transference area were used. In addition, a recirculation circuit for the extracted solution was implemented. Briefly, the *Pinus radiata* bark was ground with a double-knife mill to an average size lower than 20 mm. Then, the bark was dried at room temperature to a humidity of 24.5% (dry weight), and 100 kg (dry weight) of bark was soaked in an ethanol/water solution at a 1:20 ratio (*w*/*v*) for 120 min at 120 °C. Subsequently, the ethanol was evaporated in a vacuum (absolute pressure 0.05 bar) at room temperature. Thus, the water-insoluble particulate material after decanting and the water-soluble polyphenol fraction were obtained. Finally, the water-soluble polyphenols were lyophilized at room temperature and the obtained extracts were stored in sealed amber glass containers for further analysis.

### 2.5. Synthesis of Reduced Graphene Oxide/Nanocellulose (rGO/CNF) and Reduced Graphene Oxide/Nanocellulose/Tannin (rGO/CNF/TA) Composites

The composite material was synthesized by dissolving 100 mg of GO in 20 mL of MilliQ® water (Millipore Milli-Q, Burlington, MA, USA), stirring at 250 rpm for 15 min at room temperature. After the GO dissolution, it was put in an ultrasonic bath (Digital Ultrasonic cleaner model CD 4820, 42 kHz, 160 W, Shenzhen Codyson Electrical Co., Ltd., Shenzhen, China) with ice for 30 min. Twenty-five mL of CNF were centrifuged to 1500 rpm for 10 min to 5 °C, eliminating the supernatant. Subsequently, in a buffer Tris solution (200 mL MilliQ® water, 24.05 mg Tris Tris(hydroxymethyl)amonimethane (Merck, Santiago, Chile)), 100 mg of DA were added plus the GO solution previously prepared and the CNF, in different amounts (5, 15, 25, 50 mg), with stirring to 250 rpm and adjusting to 8.5 pH. The mix was left to react for 24 h at 60 °C and 750 rpm. The samples were cooled to 30 °C and centrifuged at 11,000 rpm for 30 min to 15 °C, eliminating the supernatant. Next, they were washed twice with MilliQ® water at 11,000 rpm for 15 min at 15 °C. The resulting samples were sonicated for 10 min and deposited in a Petri dish to dry in a vacuum oven (Huanghua Faithful Instrument Co., Ltd., Huanghua, China) at 45 °C. The samples were named rGO/CNF_5_, rGO/CNF_15_, rGO/CNF_25_, and rGO/CNF_50_.

In the case of the samples with bark tannin, the tannin was incorporated after the washing step in two proportions (12.5 and 25 mg), the mix being sonicated for 10 min and then dried as previously described. The samples were named rGO/CNF_25_/TA_5_, rGO/CNF_25_/TA_10_.

### 2.6. Physicochemical Characterization of rGO/CNF and rGO/CNF/TA Composites

The morphology and physicochemical aspects of rGO/CNF and rGO/CNF/TA composites were studied through scanning electron microscopy (SEM), Fourier transform infrared spectroscopy (FTIR), X-ray diffraction (XRD), X-ray photoelectron spectroscopy (XPS), thermogravimetric analysis (TGA), and by examining the surface charge for dynamic light scattering (DLS). A detailed characterization of the materials is included in the Supplementary Materials (SM).

### 2.7. Conductivity Measurements

The resistance values were measured with an LF meter 4192A (Keysight, Santa Rosa, CA, USA). The resistivity (ρ) could be calculated using the resistance, following Equation (1):

ρ = RS/L(1)

where R is the resistance of the sample, and S and L represent the cross-sectional area and length of the sample, respectively [28].

Thus, the conductivity (σ) was calculated by the Equation (2).

σ = 1/ρ(2)

### 2.8. Swelling Behavior of rGO/CNF and rGO/CNF/TA Composites

The swelling ratio (SR) of the materials was determined by placing samples in contact with water and phosphate-buffered saline (PBS, pH = 7.4) at room temperature for 5, 15, 30, 60, 90, 120, and 240 min. First, the samples were dried at 105 °C for 24 h and then the dry samples were cut (1.0 cm^2^), and 10 mL of liquid medium were dripped onto the surface of the samples. Each time, the excess liquid was eliminated, and the SR of the rGO/CNF and rGO/CNF/TA composites was determined using Equation (3):

(3) $$W\left(\%\right)=\frac{W_{wet}-W_{dry}}{W_{dry}}\cdot 100$$

where W_dry_ is the dry sample weight, and W_wet_ is the wet sample weight (after contact with the liquid medium).

### 2.9. Mechanical Properties

The mechanical properties of the rGO/CNF and rGO/CNF/TA composites were measured using a Universal Testing Machine (Shimadzu EZ-XS, Japan) equipped with a 20 N load cell at a temperature of 20 °C and relative humidity of 50%. All samples were cut following the shape template of 13 mm width, 19 mm length, and 0.18 thickness. The samples were held between two clamps and pulled by the top clamp at 0.1 mm/s. The elongation and breaking force were measured when the material tore apart. The elongation at break, tensile strength, and elastic modulus were calculated using Equations (4)–(6):

(4) $$Elongation\;at\;break\left(\%\right)\;=\frac{Increasing\;in\;length\;at\;breaking\;point\;(mm)}{Original\;length}\times 100\%$$

(5) $$Tensile\;strength\left(N/m^{2}\right)=\frac{Breaking\;Force\;(N)}{Cross-sectional\;area\;of\;sample\;({mm}^{2})}$$

(6) $$Elastic\;modulus\;(kPa)=Slope\times \frac{Length\;(mm)}{Cross-sectional\;area\;of\;sample\;({mm}^{2})}$$

### 2.10. Cytotoxicity Assay

Cytotoxicity was determined using the 3-(4,5-dimethylthiazol-2-yl)- 2,5-diphenyltetrazolium bromide (MTT) assay. Human dermal fibroblast cells, which were originally isolated from adult human skin tissue (Sigma, Santiago, Chile) were used to evaluate the in vitro cytotoxicity of all synthesized materials.

These experiments were conducted using a cell density of 104 cells/mL. First, 1 mL of DMEM (Genexpress, Concepción, Chile) (Dulbecco’s Modification Eagle’s Medium) medium was added to 10 mg of material and also to the individual materials (rGO and TA) to promote full contact. After 24 h of incubation at 37 °C, the supernatant was recovered and mixed with 5% (*v*/*v*) fetal bovine serum (Genexpress, Concepción, Chile (FBS) and 1% (*v*/*v*) antibiotics (100 units/mL of penicillin and 100 units/mL of streptomycin) (Genexpress, Concepción, Chile). The supernatant from each sample was added to the cells and incubated for 48 h at 37 °C under humidified air with 5% (*v*/*v*) CO_2_. At the end of the incubations, the supernatants were removed, and the cells were washed with PBS, pH 7.4. Then, 100 μL of fresh DMEM medium was added to the cells, and 5 mg/mL MTT solution was added for the determination of cell viability. The plates were incubated for 4 h at 37 °C with CO_2_, then 25 μL of the medium was removed and 50 μL of dimethyl sulfoxide (DMSO) (Genexpress, Concepción, Chile) was added to the wells. After 10 min, the supernatant was removed by aspiration, and the formazan crystals were dissolved in DMSO (100 μL per well), followed by shaking for 5 min. The absorbance was determined using a microplate reader (Biotek synergy 2, Agilent Technologies, Santa Clara, CA, USA) at a wavelength of 540 nm. The cell viability (%), relative to control cells, was calculated from A_test_/(A_control_) × 100%, where A_test_ and A_control_ are the absorbance values of the wells (with the material) and control wells (without the material), respectively. DMEM medium was used as a positive control.

### 2.11. In Vitro Wound Healing Assay (Scratch Test)

An in vitro wound healing assay was performed according to previously described experimental procedures [29], with slight modifications. Briefly, human dermal fibroblast cells (50,000 cells/well) were seeded into a 24-well plate and incubated at 37 °C for 48 h in a humidified atmosphere with 5% CO_2_. Subsequently, a vertical scratch was manually created in the middle of the human dermal fibroblast monolayer, using a 200 µL sterile pipet tip. Then, each material was fixed on CellCrown 24 inserts (Corning Incorporated, PA, USA) and placed on the 24-well plate without touching the surface. The wound closure rate and the cell migration were monitored over time (0, 12, 24, 36, 42, and 48 h) using a light microscope (MOTIC AE31, Richmond, Canada). Finally, the images were analyzed using ImageJ® software (National Institutes of Health, Bethesda, MD, USA) The wound closure rates were calculated according to Equation (5):

(7) $$Rate\;of\;wound\;closure\left(\%\right)=\frac{\left(A_{0}-A_{t}\right)}{A_{0}}\times 100$$

where A_0_ is the initial wound area and A_t_ is the wound area after each time interval.

### 2.12. Statistical Analysis

The spectra obtained in the chemical characterization of the rGO/CNF and rGO/CNF/TA composites were acquired in triplicate and processed using OriginPro 8.5® software (OriginLab Corporation, MA, USA). All experiments were carried out in triplicate, and the results are expressed as the mean ± standard deviation value. The studies of means were performed using multifactorial analysis of variance (ANOVA), whose accepted significance was *p*-value ≤ 0.05, and an analysis of multiple ranges (Tuckey test, 95% confidence) in Statgraphics Centurion XVII® software (Statpoint Technologies. INC., Virginia, USA). The mean values and the error bars are reported in each figure.

## 3. Results and Discussion

### 3.1. Morphological Characterization of rGO/CNF and rGO/CNF/TA Composites

**Figure 2:**
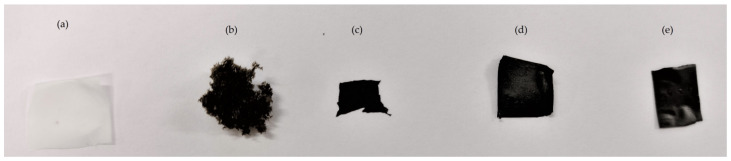
Images of (**a**) CNF, (**b**) GO, (**c**) rGO, (**d**) rGO/CNF_25_, (**e**) rGO/CNF_25_/TA_10_.

The raw and the composite materials formulated are presented in Figure 2. After the reduction with DA, rGO (Figure 2c) presented a homogeneous aspect, black in color, shapeable and easy to mold, different from GO (Figure 2b). After the addition of CNF (Figure 1a), no visual changes were observed (comparing Figure 2c vs. 2d) or with TA addition (comparing Figure 1c vs. 1d vs. 1e) in the micromorphology.

**Figure 3:**
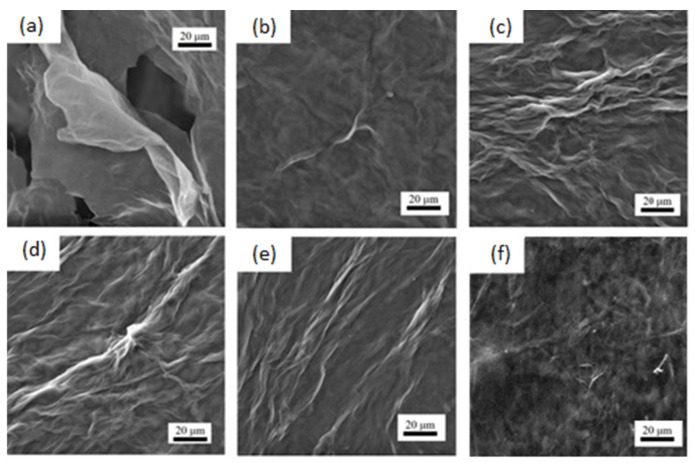
SEM images (**a**) GO, (**b**) rGO/CNF_5_, (**c**) rGO/CNF_15_, (**d**) rGO/CNF_25_, (**e**) rGO/CNF_50_ (**f**) rGO/CNF_25_/TA_10_.

The morphology of the samples was characterized by scanning electron microscopy (SEM) measurements. In Figure 3, the GO presented an unordered sheets distribution exhibiting a wrinkled structure, which was due to the freeze-drying process during which the stacked GO layers were further separated from each other, promoting a significant increase in the specific surface area. After the conformation of the composite, the GO wrinkled structure disappeared and the surface presented an increased number of fibers, which is concordant with the amount of CNF added (Figure 3b–e). The TA inclusion softened the surface even more (Figure 3f), making it more homogeneous, coating and connecting with CNF and rGO. A structural comparison with other composite materials based on rGO/CNF led to a similar conformation: the GO wrinkles soften with the CNF addition [30,31].

### 3.2. Spectroscopical Characterization of rGO/CNF and rGO/CNF/TA Composites

**Figure 4:**
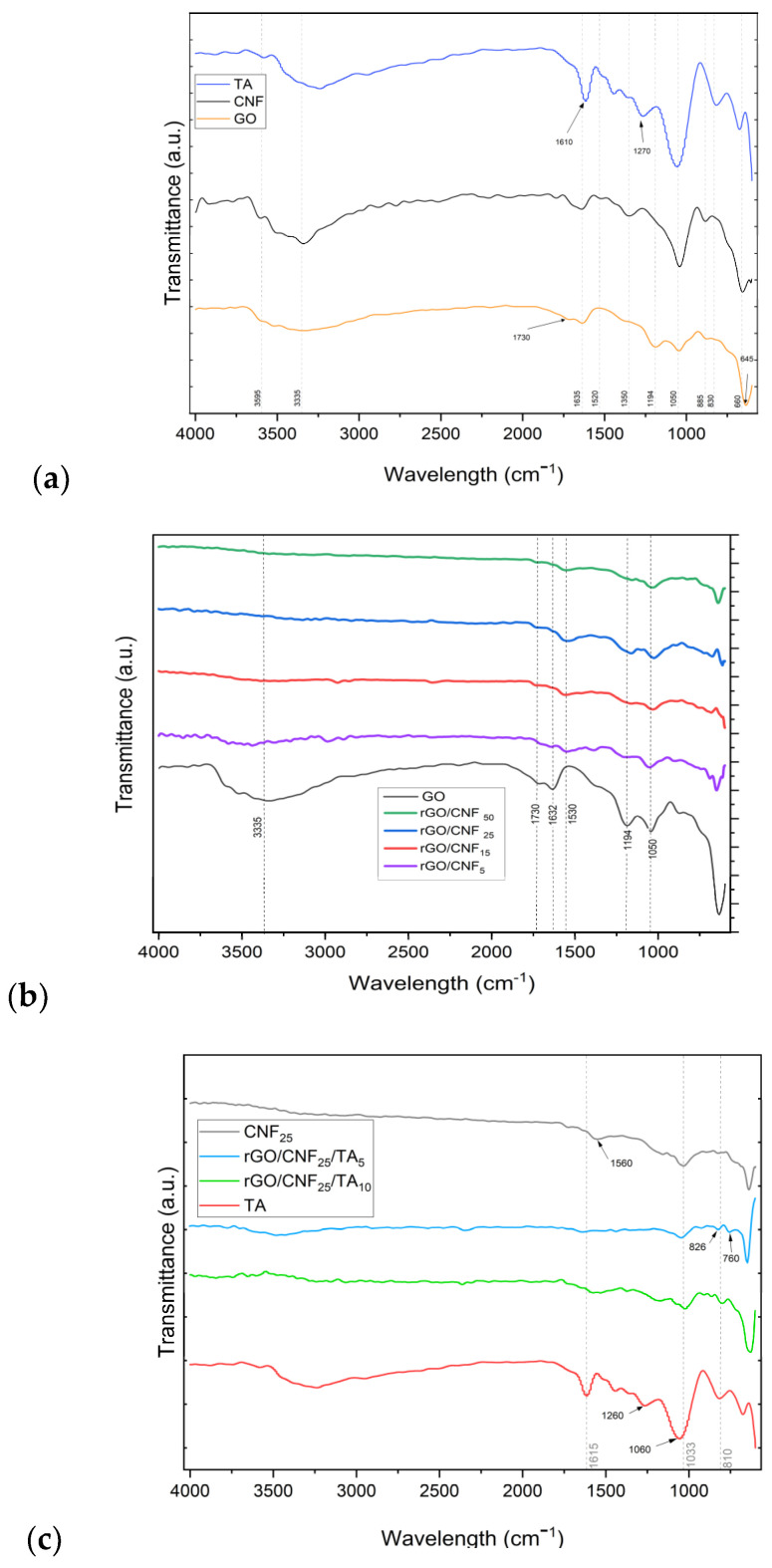
FTIR Spectra (**a**) GO, CNF, TA, (**b**) rGO and rGO/CNF_5_, rGO/CNF_15_, rGO/CNF_25_, rGO/CNF_50_ samples, (**c**) TA, rGO/CNF_25_, rGO/CNF_25_/TA_5_, rGO/CNF_25_/TA_10_.

The FTIR GO spectrum (Figure 4a) shows a characteristic band at 1635 cm^−1^, which arises from C=C stretching vibration of the sp^2^ carbon skeletal network (from unoxidized sp^2^ C=C bonds) [32,33]. The bands related to oxygen-containing functional groups were visible, such as the C–OH stretching vibration at ∼3335 cm^−1^, carbonyl groups –C=O at 1730 cm^−1^, C–O–C epoxy groups at 1194 cm^−1^, and C–O flexion vibrational mode at 1050 cm^−1^ [34]. The CNF spectrum presented a band at 3333 cm^−1^ related to O–H stretching vibration, at 1635 cm^−1^ for –OH bending of absorbed water and at 1031 cm^−1^ for C–O stretching and at 885 cm^−1^ for –CH alkane bending [33]. The TA spectrum presented a band at 3200 cm^−1^ for the aromatic and non-aromatic hydroxyl groups, at 1615 cm^−1^ the C=O stretching vibration, at 1260 and 1060 cm^−1^ the C–O aromatic stretching, and at 1030 cm^−1^ the aliphatic C–O stretching [35,36].

The FTIR demonstrated the successful reduction of GO by DA (Figure 4b). The manifold peaks within the range of 800 to 1900 cm^−1^ were attributed to various oxygen-containing groups on the surface of the GO sheet and CNF, which after DA reduction treatment were modified. In the composite rGO/CNF_5_, rGO/CNF_15_, rGO/CNF_25_, and rGO/CNF_50_, the C=O stretching vibration of the carboxyl group and epoxy deformation peak present at 1730 cm^−1^ and 1194 cm^−1^, respectively, almost disappeared, and the band at 1050 cm^−1^ was reduced, indicating that the oxygen-containing functional groups of GO were successfully removed (Figure 4b). During the grafting of CNF to the GO, the intensity of the absorbed water (at 1632 cm^−1^) become substantially reduced, while the C=C stretching at 1559 cm^−1^ emerged. The FTIR spectra for rGO/CNF_5_, rGO/CNF_15_, rGO/CNF_25_, and rGO/CNF_50_ seem identical, suggesting analogous functional groups constituents. The TA inclusion reduced even more main functional groups of rGO/CNF_25_; in the samples rGO/CNF_25_/TA_5_ and rGO/CNF_25_/TA_10_, the vibrations 1732, 1559, 1030 cm^−1^ almost disappear (Figure 4c).

**Figure 5:**
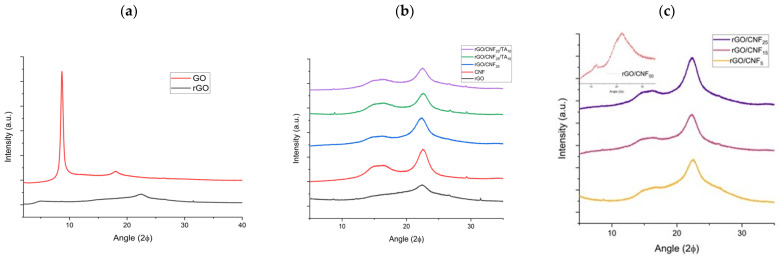
(**a**) X-ray diffraction patterns of GO and rGO, (**b**) CNF, rGO composite materials rGO/CNF_25_, rGO/CNF_25_/TA_5_, rGO/CNF_25_/TA_10_, and (**c**) rGO/CNF_5_, rGO/CNF_15_, rGO/CNF_25_, rGO/CNF_50_.

The reduction of GO and the effect of grafting CNF and TA was also verified by XRD and XPS spectroscopy. Figure 5 presents the XRD patterns of GO, rGO, CNF, and the composite materials, including the samples with TA. GO presented a single peak at 2θ = 8.68°, which corresponds to an interlayer d-spacing of 10.2 Å (Figure 5a). This larger d-spacing may be due to the presence of oxygen functionalities such as epoxy and hydroxyl on the basal planes and carboxyl on the edges of the graphene sheets. After the GO reduction by DA, a new broad peak at 2θ = 22.47° was obtained, reducing the inter-planar distance to 3.95 Å, which may be attributed to the π-π stacking interactions of the aromatic rings of the coated PDA and/or rGO [37], indicating the successful GO reduction [38]. CNF presented a typical signature of cellulose I structure, with peaks at 16.26° and 22.57° (Figure 5b) [39]. The CNF inclusion on the sheet preserved the peaks, and the crystallinity increased until 15% CNF (Figure 5c). This value rose with TA addition, caused maybe for the structuration given for the major number of interactions. 

**Figure 6:**
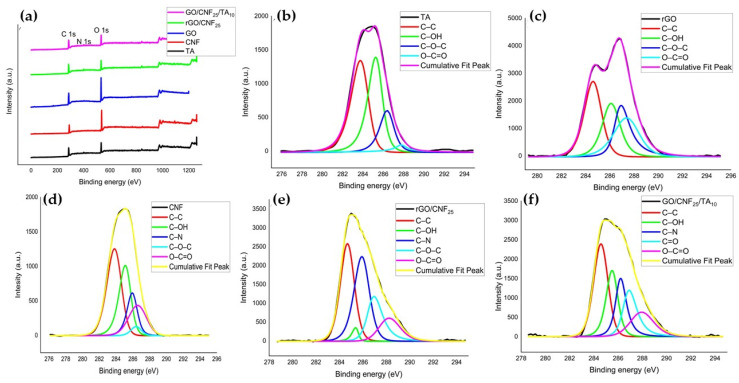
(**a**) XPS survey spectrum of GO, TA, CNF, rGO/CNF_25_, rGO/CNF_25_/TA_10_; deconvoluted C 1S XPS spectra of (**b**) TA, (**c**) rGO, (**d**) CNF, (**e**) rGO/CNF_25_, (**f**) rGO/CNF_25_/TA_10_.

The surface compositions of GO, CNF, TA, rGO/CNF_25_, and GO/CNF_25_/TA_10_ were studied by XPS (Figure 6). As can be seen in Figure 6a, all samples exhibited two characteristic peaks in 284.8 eV corresponding to the C 1s and 533.6 eV corresponding to the O 1s coinciding with data reported by Liu et al. [40]. The characteristic peak 401 eV corresponding to the N 1s appeared in the spectrum of TA, rGO/CNF_25_, and GO/CNF_25_/TA_10_. Additionally, for the samples rGO/CNF_25_ and rGO/CNF_25_/TA_10_, the C/O ratio increased markedly due to the elimination of oxygen-containing functional groups, indicating the successful reduction of GO.

Deconvolution spectra at the basic C 1s level of all samples shown in Figure 6b–e, and 6f resulted in four maximum components with binding energies at 284.8, 285.6, 286.6, and 287.6 eV for the C–C, C–OH, C=O, and O–C=O species, respectively. The samples containing CNF at 286.1 eV evidenced the species C–N (Figure 6d–f), increasing the intensity of a C–N bond in the GO presence (Figure 6e), indicating that the GO has reacted with the DA to form a new covalent bond. This showed that the CNF was successfully functionalized with catechol groups through PDA coating. Similar results were reported by other authors working with similar materials [41]. The TA inclusion decreases the C–N intensity, which can be attributed to a higher C–OH interaction from the TA compound’s structure.

The percentage in area of the maximum components with binding energies of all the samples in the deconvolution of the spectra at basic level C 1 s is presented in Table 1. All samples had the aliphatic group (C–C%) with high percentages, this being the case of TA, CNF, and GO/CNF_25_/TA_10_, with more than 45% of the area. Comparing the behavior of the alcohol, amine, and ester groups of CNF and GO with rGO/CNF_25_, there is a clear reduction in their % of the area observed, caused by the formation of a new carbonyl group.

The rGO/CNF_25_ loaded with the TA increases the C–OH bond until reaching values close to 30% in the peak area. The amide, carboxyl, and C–N groups exhibit similar behavior, which may be because these groups are attached to the carbon skeleton of CNF or have been able to form hydrogen bonds with GO.

### 3.3. Thermal Stability of the Composite Materials

The thermal stability of CNF, rGO, and the rGO/CNF_25_, GO/CNF_25_/TA_10_ composite film was evaluated via thermogravimetric analysis (TGA) and differential thermogravimetric analysis (DTG) (Figure 6a and Figure S1, see in Supplementary Materials, respectively). Based on TGA and DTG curves the characteristic temperatures were determined on Table 2. CNF initial weight losses starting at 168.27 °C, showed a main weight loss at 226.79 °C, which was attributed to the decomposition of cellulose [42]. The rGO presented two stages in the process of thermal weight loss: the first stage ended at 100 °C with 10 wt% weight loss, due to the moisture, and the second stage initiated at 314.04 °C and reachied a maximum at 345.87 °C, which was mainly attributed to the decomposition of the labile residual oxygen-containing groups on the surface of GO [43]. Thus, rGO was thermically more resistant than CNF. The addition of 25% CNF to rGO decreases the T_i_ to 171.63 and T_max_ to 223.93 °C, respectively. The presence of hemicellulose and lignin, which decompose at low temperatures, caused a reduction of thermal stability on the composite material. Other studies have shown a similar effect on cellulose/graphene composites: the inclusion of more CNF content lowering the thermal decomposition temperature [44]. The further addition of TA to the composite reduced the thermal stability even more, going down the onset temperature at 153.13 °C and T_max_ at 202 °C, which can be associated to the physical interaction interactions between the composite’s components—as was observed in the chemical analysis—decreasing the rGO stability. The residual content carbon reaches the maximum for the sample containing TA, since the contribution of carbon skeletal content in the phenolic structure which is coincident with XPS analysis.

### 3.4. Conductivity, Surface Charge and Wettability of the Composite Materials

**Figure 7:**
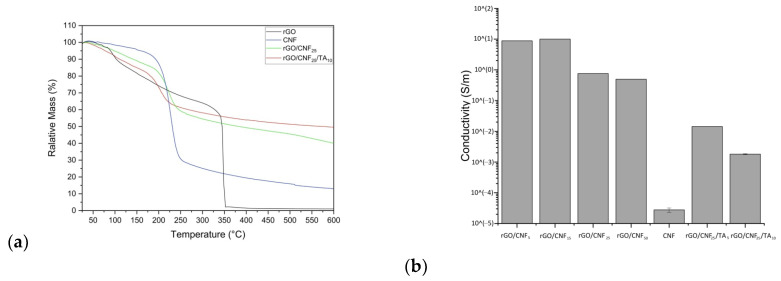
(**a**) Thermogravimetric analysis (TGA) of rGO, CNF, rGO/CNF_25_, rGO/CNF_25_/TA_10_, and (**b**) conductivity of rGO/CNF_5_, rGO/CNF_15_, rGO/CNF_25_, rGO/CNF_50_, CNF, rGO/CNF_25_/TA_5_, and rGO/CNF_25_/TA_10_.

A four-probe tester was used to evaluate the surface electrical resistance of the composite materials. In Figure 7b, the conductivity of rGO with different amounts of CNF is shown, also rGO/CNF_25_ with different amounts of TA. The GO after the reduction with PDA was a fragile material—as will be discussed further—making it impossible to measure it (data not shown). The incorporation of increasing amounts of CNF decreased the electrical activity of the materials, which can be mainly attributed to the insulating nature of the CNF [45]. Neat CNF was not electrically conductive. When the amount of CNF was increased, their absorbance on the surface of rGO increased, which leads to the thickening of the CNF stacking structure units, reducing the connection of rGO sheets and eliminating the conductive channels, thus diminishing the electrical activity. The TA addition resulted in an inverse effect on the material’s conductivity, which can be attributed to the fact that TA exert a kind of blocking of the electrical charges. The TAs have several alcohol groups in their structure that do not allow the molecule to polarize, thus preventing the movement of electrons and the conductivity per se.

The surface charge of CNF and GO was −53.3 mV and −96.9 mV, respectively (Table S3, see in Supplementary Materials). When the CNF was added to the GO to form the composite materials, this value was reduced to −31.95 mV on average, and no significant difference was observed among samples. The value diminution may be caused by the assembly of the functional compounds after the DA reduction. The TA addition increased the *z*-value of composite material, making it more negative (rGO/CNF_25_/TA_5_: −85.2 mV and GO/CNF_25_/TA_10_: −89.7 mV), which may be caused by the contribution of –OH groups of flavan-3-ols, which is in concordance with the conductivity behavior of TA samples. The z-values found for CNF and GO were comparable to those reported by other authors [46,47]. Reports of the z-potential of the other materials CNF/rGO/TA were not found.

The distilled water angles of the CNF, GO, rGO/CNF_5_, rGO/CNF_15_, rGO/CNF_25_, and rGO/CNF_50_ was determined as a measure of the material’s wettability (see in Supplementary Materials). CNF (46.6°) was more hydrophilic than GO (61.8°), caused by the availability of functional groups on CNF. The addition of CNF to the samples resulted in a decrease in the number of oxygens, increasing the hydrophobicity of the material, augmenting the contact angle in direct proportion to CNF added, having an angle of 89.4° the sample with 50% CNF. Similar effects were observed by Miao et al. [48], working with microcrystalline cellulose added to GO. TA addition reduced the contact angle to 84.3° (rGO/CNF_25_/TA_5_) and 72° (rGO/CNF_25_/TA_10_), which can be related to TA’s hydrophobic nature, caused by the residual functional groups being unreacted in the composite.

### 3.5. Swelling Behavior of Composite Materials

The swelling percentage of the materials was carried out using PBS as a model fluid in order to determine the absorption capacity over time (Figure S2, see in Supplementary Materials). The material initial moisture content was on average 11.8% (Table S4, see in Supplementary Materials). The CNF, by their hydrophilic nature, absorbed the fluid instantaneously, reaching 100% of swelling capacity in the first minutes (data not shown). The rGO had a slow absorption rate, reaching a maximum capacity of 48 g/g in close to 80 min. The CNF incorporation into rGO helped to augment the velocity of PBS absorption in the first minutes, but the equilibrium values were maintained at close to 50 g/g in less time than rGO. The TA inclusion helped even more to improve the material’s swelling velocity (25 g/g in 5 min), which may be caused by the availability of OH groups, contributing to the interaction with the fluid, since PBS has a high hydrogen bond acceptor value.

### 3.6. Tension and Deformation of Composite Materials

**Figure 8:**
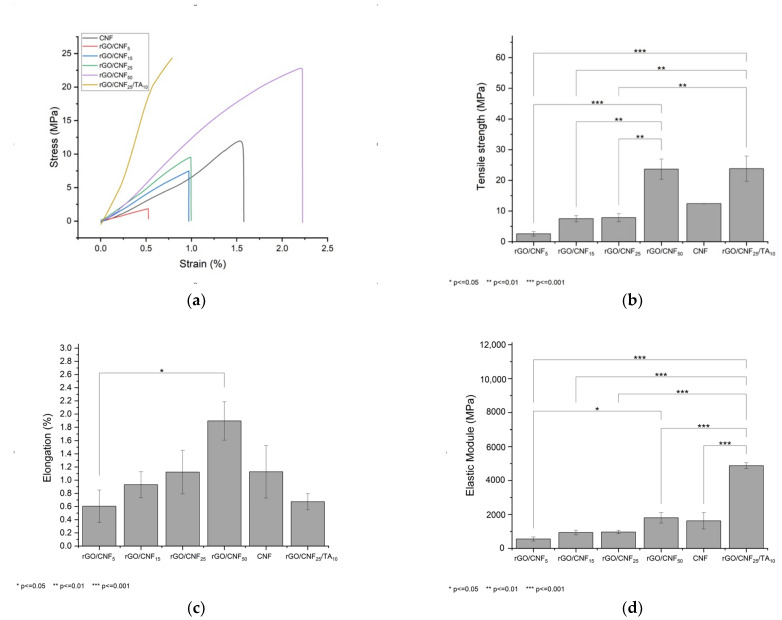
Mechanical properties of materials including (**a**) stress vs. strain curves, (**b**) tensile strength, (**c**) elongation at break, and (**d**) elastic modulus of the neat CNF, rGO, and rGO/CNF_5_, rGO/CNF_15_, rGO/CNF_25_, rGO/CNF_50_, rGO/CNF_25_/TA_10_.

Figure 8a presents the effect of adding different CNF concentrations on the mechanical properties of the material. The CNF presented a tensile strength of 12.4 MPa, a maximum deformation of 1.13% and an elastic module of 1624 MPa. The rGO when synthesized was extremally fragile and it was not possible to analyze it mechanically. The addition of CNF allowed that tensile tests can be performed, increasing the tensile strength at a higher CNF concentration; thus, samples rGO/CNF_5_, rGO/CNF_15_, and rGO/CNF_25_ show tensile strength of 2.6 MPa, 7.5 MPa, and 7.8 MPa, respectively (Figure 8b). The sample with notable resistance was rGO/CNF_50_ (23.6 MPa). The same behavior was observed for the elongation and elastic module of the samples (Figure 8c,d), reaching in the most favorable case (rGO/CNF_50_) an elongation at break of 1.9% and elastic modulus of 1806 MPa. These results are probably caused by CNF interactions with the oxygenated groups of GO through hydrogen bonds, promoting the interfacial joint and improving the mechanical properties [4].

The TA inclusion in the sample with CNF 25% (rGO/CNF_25_/TA_10_) showed an even higher improvement in the tensile strength, (7.8 MPa rGO/CNF_25_ vs. 23.8 MPa rGO/CNF_25_/TA_10_), as well as elongation at a break of 0.6% and an elastic modulus of 4879 MPa. TA would produce π-π interactions with the graphene sheets [49]. Additionally, TA can interact with cellulosic surfaces in multiple ways. The basic tannin moieties, the phenolics, present significant H-bonding capacity, enabling it to interact strongly with practically any hydrophilic substrate [50]. Thus, besides H-bonding and quadrupolar, electrostatic interactions are favored between tannin and negatively charged cellulose surfaces [51]. Therefore, the increase of the material’s mechanical properties is caused by TA/rGO/CNF interactions.

### 3.7. Cytotoxicity Assays

**Figure 9:**
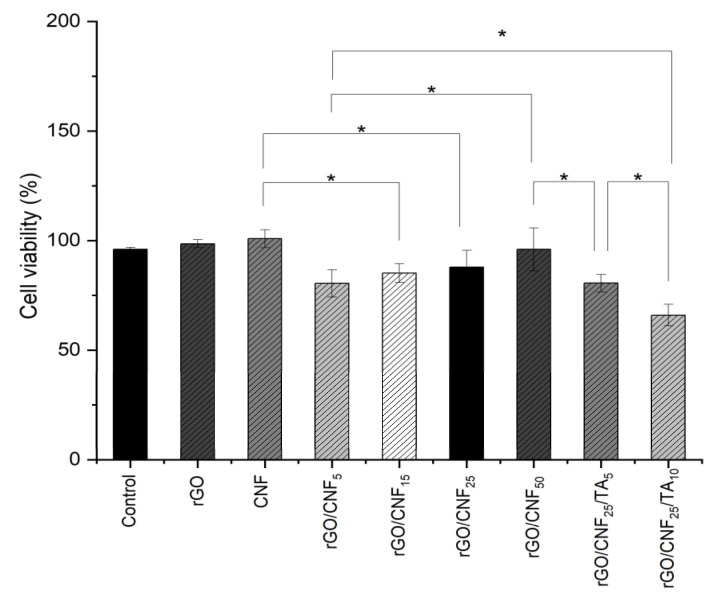
Human fibroblast cell viability in presence of neat CNF, rGO and rGO/CNF_5_, rGO/CNF_15_, rGO/CNF_25_, rGO/CNF_50_, rGO/CNF_25_/TA_5_, rGO/CNF_25_/TA_10_. The asterisk indicate a significant difference (* *p* < 0.05) when analyzed by Tuckey test by one-way ANOVA analysis.

An ideal wound dressing should exhibit biocompatible properties and not produce intolerable toxic effects on the body. Biocompatibility of composite materials was examined through cell viability using human fibroblast as a model cell (Figure 9). The raw CNF viability was similar than rGO. The addition of CNF to the composite increased the compatibility, proportionally to the CNF amount added. The TA inclusion was not favorable, decreasing the cell viability, especially for the higher concentration studied. Nevertheless, the cell viability for the highest TA concentration was higher than 65%, whereas an optimal cellular viability of 80% was determined for the sample rGO/CNF_25_/TA_5_. It is well known that TA exert several bioactivities such as anti-inflammatory, antimicrobial, antioxidant, and anticancer activities, in addition to their involvement in cardiovascular, neuroprotective, and general metabolic disease prevention [18]. Thus, we believe that the TA inclusion in the wound dressing will contribute to improving healing. Nevertheless, TA have also been reported to be toxic for the cells, depending on their chemical structure [52]. The TA studied here were obtained from pine bark, and their chemical composition is mainly condensed tannins with high molecular weight (see Tables S1 and S2 in Supplementary Materials). Studies focusing on pine radiata and human fibroblast to evaluate cytotoxicity were not found, but other authors working with Enzogenol®, also a pine radiata bark extract, evaluated the influence of different concentrations of extracts (30–5000 ng/mL) on human neuroblastomas cells to determine cell survival and did not find significant changes [53]. In our cytotoxicity test after 24 h of material incubation, the released TA reached values of 53,500 ng/mL for 5% TA and 93,500 ng/mL for 10% TA, which was almost twenty times superior to the concentration assayed in that report.

### 3.8. In Vitro Wound Healing Assay (Scratch Test)

**Figure 10:**
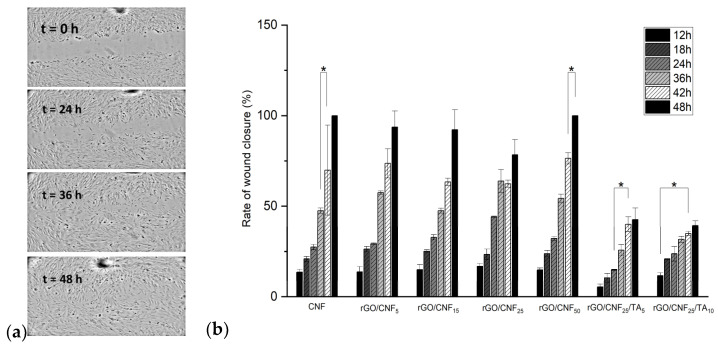
(**a**) Wound closure images on times 0, 24, 36, and 48 h for rGO/CNF_50_; (**b**) in vitro wound healing over time for raw CNF, rGO/CNF_5_, rGO/CNF_15_, rGO/CNF_25_ rGO/CNF_50_ rGO/CNF_25_/TA_5_, rGO/CNF_25_/TA_10_. The asterisk indicate a significant difference (* *p* < 0.05) when analyzed by Tuckey test by one-way ANOVA analysis.

An in vitro wound healing assay (scratch) using human fibroblast cells was performed. In this test, it was assumed that fibroblast cells would attempt to migrate along the edges of the scratch zone to establish cell-cell contact, leading to the closure of the wound. Both cell migration and wound closure rates were monitored over time (Figure 10 and Figure S3 in Supplementary Materials). The optical microscopy images show that fibroblast cells migrated to the scratch zone after 48 h incubation in contact with the materials (Figure 10a), which was confirmed by the wound closure rates (Figure 10b). The samples rGO/CNF_5,_ rGO/CNF_15_, and rGO/CNF_25_ had a similar wound closure rate. The wound closure of the sample rGO/CNF_50_ was similar to the control, maybe influenced by the higher amount of CNF. The TA inclusion in the composite was not favorable for the migration of cells, obtaining values lower than those of the samples without TA, which agrees with cell viability results. Schmit et al. [54] studied different bark fractions (rich in catechins) of *P. rigida*. Preparations from this bark are used in traditional medicine because of their anti-inflammatory, astringent, expectorant, antidiarrheic, antihemorrhagic, antimicrobial, and wound-healing properties. They found through the scratch assay that the migratory and proliferative activities of mouse fibroblast after 12 h of incubation were dose-dependent. Most samples showed enhanced cell numbers when concentrations of 1 and 10 μM were studied, whereas 20 μM concentrations led mostly to a reduction. Additionally, from the eleven compounds tested, only epicatechin-3-*O*-gallate and 4′-*O*-methylepicatechin-3-*O*-gallate had a favorable effect on wound healing. If the chemical composition of our bark pine extracts is compared with those results, in their composition gallate moieties were not found (see in Supplementary Materials), which could explain the unfavorable results observed.

## 4. Conclusions

A new composite material based on rGO/CNF/TA was developed by physical crosslinks and PDA reduction was achieved. The CNF increasing incorporation into rGO led to a reduction in thermal stability, surface charge, and conductivity, but the mechanical properties improved. The TA were released from the composite material. The material’s cytotoxicity and wound healing on human fibroblasts improved with the CNF incorporation, but the TA addition resulted in a slight diminution of the cell viability and reduced cell migration, which may be associated with the doses used and the chemical nature of pine extracts. However, the sample rGO/CNF_25_/TA_5_ showed a cell viability of 80%. It can be concluded that synthetized materials might be used as efficient dressing for wound healing. Further in vivo tests will be carried out in order to confirm these in vitro results.

## Figures and Tables

**Table 1:** Percentage in area of maximum components with binding energies of all samples.

Sample	C–C%	C–OH%	C–O–C%	O–C=O%	C–N%
TA	47	33	16	3	N.D.
CNF	46	32	17	3	19
GO	30	23	22	23	N.D.
rGO/CNF_25_	38	2	37	21	13
GO/CNF_25_/TA_10_	46	28	25	25	20

N.D.: not detected.

**Table 2:** Thermal analysis data for CNF, rGO and composite materials.

Sample	T_i_ (°C) ^1^	T_max_ (°C) ^2^	T_b_ (°C) ^3^	M_600_ (%) ^4^
CNF	168.27	226.79	269.70	13.84
rGO	314.04	345.87	381.39	1.33
rGO/CNF_25_	171.61	223.93	267.44	32.10
rGO/CNF_25_/TA_10_	153.13	202.24	248.01	47.62

^1^ T_1_: the extrapolated onset temperature of the thermal decomposition peak (also the extrapolated onset temperature of the derivative DTG curve, ^2^ T_max_: the temperature at the maximum weight loss rate, ^3^ T_b_: the extrapolated final temperature of the DTG curve. ^4^ M_600_: the residual carbon content at 600 °C.

## Data Availability

Not applicable.

## Supplementary Materials

The following supporting information can be downloaded at: https://www.mdpi.com/article/10.3390/polym15122752/s1, Figure S1. Derivative thermogravimetric analysis r GO, CNF, rGO/CNF_25_, rGO/CNF_25_/TA_10_; Figure S2. Swelling behavior of neat materials and composite samples on PBS fluid on time; Figure S3. Migration of fibroblast cells in Scratch test (wound healing) for several samples; Table S1. Phenol composition and content of the *Pinus radiata* bark extract; Table S2. Average molecular weight number (Mn) of pine extracts at different percentages of sample development, determined by GPC. Table S3: Surface charge and contact angle of the samples. Table S4: Initial moisture content of the samples.
