# Supplementary material for: Self-Assembled CNF/rGO/Tannin Composite: Study of the Physicochemical and Wound Healing Properties
Source: Polymers (Basel). 2023 Jun 20;15(12):2752. doi: 10.3390/polym15122752 (PMC10304054; doi:10.3390/polym15122752)
Supplement: Supplementary file 1 [file polymers-15-02752-s001.zip › polymers-2366275-supplementary.pdf]

Supplementary Materials

Self-Assembled CNF/rGO/Tannin Composite: Study of the Physicochemical and Wound Healing Properties

Katherina Fernández [1,*], Aylen Llanquileo [1], Monserrat Bustos [1], Valentina Aedo [1], Isleidy Ruiz [1], Sebastián Carrasco [1], Mauricio Tapia [1], Miguel Pereira [2], Manuel F. Meléndrez [3], Claudio Aguayo [4] and Leonard I. Atanase [5,6,*]

1. Laboratorio de Biomateriales, Departamento de Ingeniería Química, Facultad de Ingeniería, Universidad de Concepción, Concepción 4070386, Chile; ayllanquileo@udec.cl (A.L.); monserratbustos@udec.cl (M.B.); vaedo2017@udec.cl (V.A.); isruiz@udec.cl (I.R.); scarrasco2017@udec.cl (S.C.); mtapia2017@udec.cl (M.T.)
2. Laboratorio de Productos Forestales, Departamento de Ingeniería Química, Facultad de Ingeniería, Universidad de Concepción, Concepción 4070386, Chile; miguelpereira@udec.cl
3. Grupo Interdisciplinario de Nanotecnología Aplicada (GINA), Laboratorio de Materiales Híbridos (HML), Departamento de Ingeniería de Materiales (DIMAT), Facultad de Ingeniería, Universidad de Concepción, Concepción 4070386, Chile; mmelendrez@udec.cl
4. Departmento de Inmunología y Bioquímica Clínica, Facultad de Farmacia, Universidad de Concepción, Concepción 4070386, Chile; caguayo@udec.cl
5. Faculty of Medical Dentistry, Apollonia University of Iasi, 700511 Iasi, Romania
6. Academy of Romanian Scientists, 050045 Bucharest, Romania

* Correspondence: kfernandeze@udec.cl (K.F.); leonard.atanase@yahoo.com (L.I.A.)

1. Materials and Methods

1.1 Materials

Graphite powder (flakes; mesh 325) was purchased from Asbury Online (Asbury Carbons, New Jersey, USA). Dopamine hydrochloride ($DA - HCl$, ≥ 98.0%) was purchased from Sigma-Aldrich Company (St. Louis, MO). The other chemicals and solvents, such as tris(hydroxymethyl)aminomethane (buffer Tris), sulfuric acid (H2SO4, 98%), phosphoric acid (H2PO3, 85%), oxygenated water (H2O2, 60 vol), silver nitrate (AgNO3, 0.1 N), potassium permanganate powder (KMnO4, 99.9%) and hydrochloric acid (HCl, 37% v/v), were purchased from Merck (Darmstadt, Germany). These chemicals were used as received, without further purification. Milli-Q® water and distilled water were used throughout the study. Cellulases Quimizime B was provided by CHT group (Santiago, Chile).

1.2. Material characterization

X-ray diffraction (XRD). The X-ray diffraction (XRD) was used to determine the oxidation degree of GO and the crystallinity of rGO/NCF and rGO/CNF/TA composites. The measurements were carried out on the X-ray diffractometer (Bruke Axs, D4 Endeavor, USA) with reference target: Cu Kα radiation (λ=1,541841 Å; 2,2 kW), voltage: 40 kV, and current: 20mA. The samples were measured from 2 to 50° during 141 s with steps of 0.02°.

Fourier Transform Infrared Spectroscopy (FTIR). The FTIR was used to investigate the chemical nature of interaction of rGO/NCF and rGO/CNF/TA composites. The spectra were recorded in the Perkin Elmer UATR Two FTIR Spectrometer. The wavenumber range analyzed was 4000-500 cm-1 and a total of 40 accumulated scans were acquired.

X-ray photoelectron spectroscopy (XPS). XPS technique was used to quantitatively identify the surface chemistry of rGO/NCF and rGO/CNF/TA composites, also the raw components NCF, TA and rGO were analyzed. The measurements were carried out in a Surface Analysis Station 1 (STAIB model RQ300/2, USA) at ultravacuum conditions (< 10-9 bar) equipped with a hemispherical electron analyzer (SPEC PHOIBOS 100, Germany). The photoelectrons were excited with non-monochromatic radiation Mg Kα (1486.6 eV) and analyzed with a constant energy step of 1 eV. The X-ray source was used with a strength of 300 W.

Thermogravimetric analysis (TGA). TGA technique was used to evaluate the thermal stability of gelatin, GO and rGO/NCF and rGO/CNF/TA composites. The measurements were carried out in a Cahn-Versatherm thermogravimetric analyzer with sensitivity of 0.1 μg, heating rate of 10°C/min under nitrogen atmosphere (100 mL/min) and a temperature range from 30 °C to 800 °C.

Scanning electron microscopy (SEM). The SEM analysis was used to investigate the micromorphology of rGO/NCF and rGO/CNF/TA composites. SEM images were recorded using a JEOL JSM-6380LV, Japan model microscope at 10 kV. The aerogels were coated using a gold sputter coater and their surfaces were observed at different resolutions. In addition, the SEM images were processed using ImageJ® software to determinate the average pore sizes.

Surface charge measurements. The surface charge was determined through ζ-potential measurements using the Dynamic Light Scattering principle (SZ-100 Nano particle analyzer, Horiba Scientific, Japan). The measurements were carried out for the GO and gelatin-GO aerogels. Samples 1.0 cm^3 in volume were dissolved in Milli-Q® water pH 6.5, shaken and sonicated for 20 min to achieve homogeneity. Finally, the samples were measured in triplicate.

Wettability. A contact angle test was obtained with a Dataphysics-OCA20 instrument (Dataphysics, Filderstadt, Germany).

Figures:

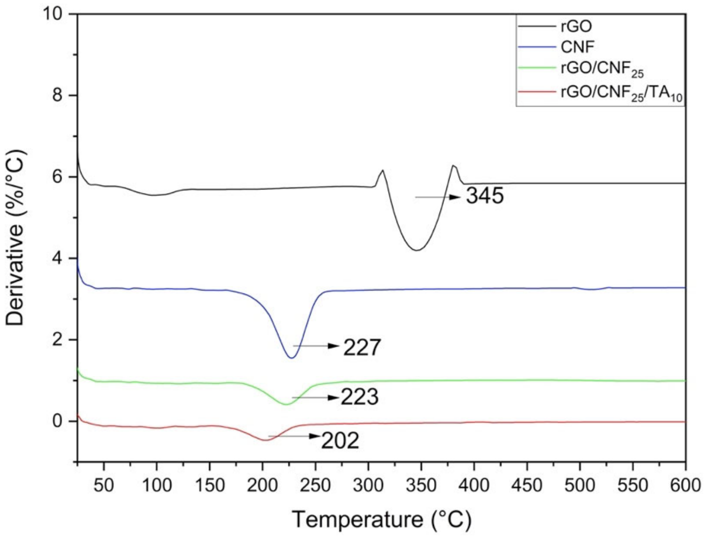

Figure S1: Derivative thermogravimetric analysis (DTG) of rGO, CNF, rGO/CNF_{25} and rGO/CNF_{25}/$TA_{10.}$

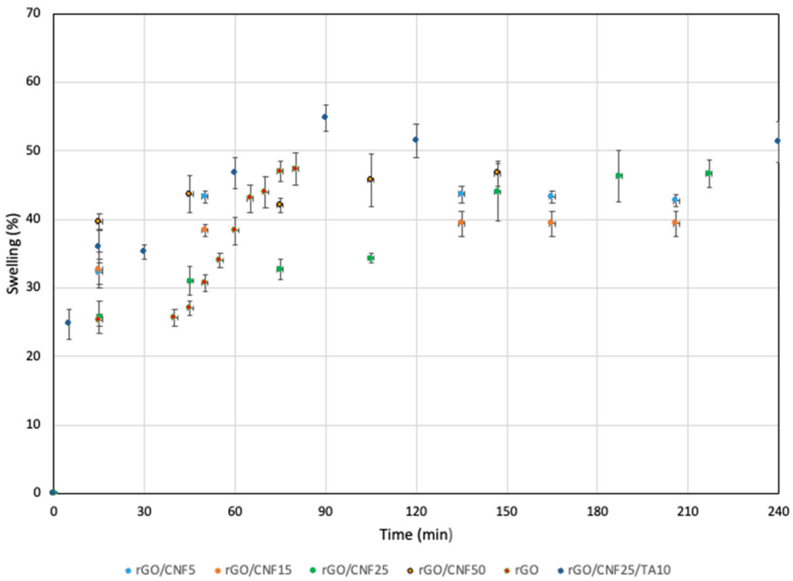

Figure S2. Swelling behavior of neat materials and composite samples on PBS fluid on time.

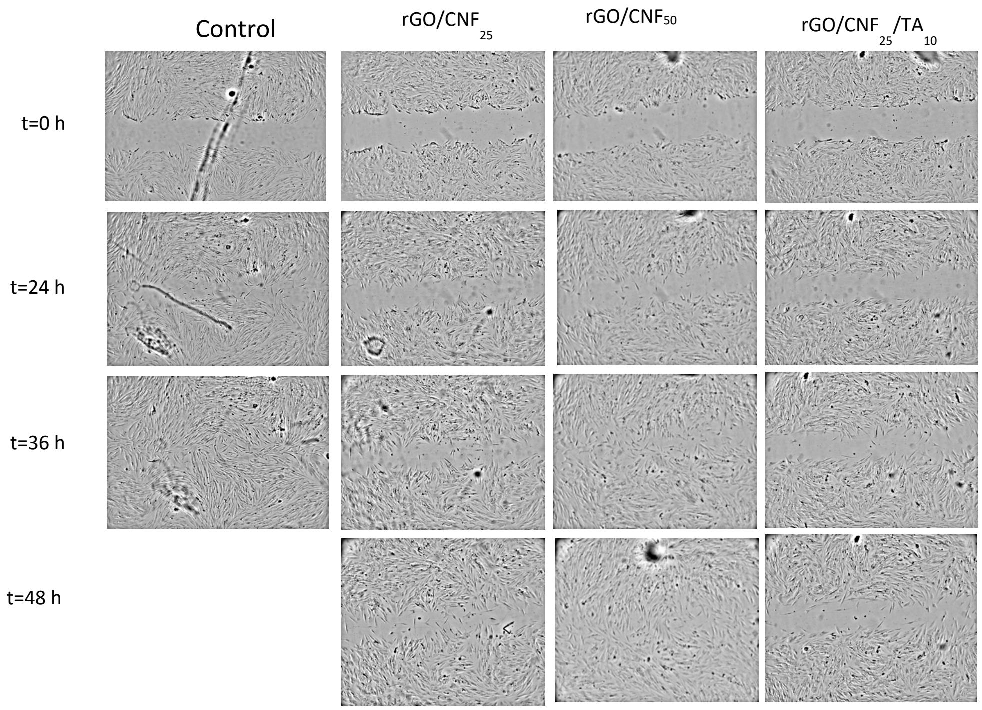

Figure S3: Migration of fibroblast cells in Scratch test (wound healing) for several samples

Table S1. Phenol composition and content of the *Pinus Radiata* bark extract

Compounds	Content (mg per gram of extract)
(-)-Catechin	13.8
Taxifolin	13.9
p-Hydroxybenzoic acid	7.6
Homovanillic acid a	6.7
Quercetin	4.3
Proanthocyanidin *B-2*	3.3
(+)-Epicatechin	2.8
Dihydroxybenzoic acid b	2.7
Dihydroxybenzoic acid b	1.8
Syringic acid a	1.1
3,4-dihydroxyphenyl acetic acid	0.9
Dihydroxybenzoic acid b	0.2
Epigallocatechin	n.d.

n.d.: not detected, a: tentatively identified compounds, b: not recognized isomers.

Table S2. Average molecular weight number (Mn) of pine extracts at different percentages of sample development, determinate by GPC.

% sample development	Mn
10	438
25	749
50	1355
100	63277

Table S3. Surface charge and contact angle of the samples

Sample	Surface charge (mV)	Contact Angle (°)
CNF	-53.3 ± 1.7	46.6 ± 0.9
GO	-96.9 ± 3.8	61.8 ± 0.6
rGO/CNF_5	-33.9 ± 3.1	70.5 ± 2.9
rGO/CNF_{15}	-33.7 ± 2.7	73.9 ± 2.5
rGO/CNF_{25}	-28.1 ±2.1	83.8 ± 2.9
rGO/CNF_{50}	-32.1 ± 1.4	89.4 ± 2.5
rGO/CNF_{25}/TA_5	-85.2 ± 0.5	84.3 ± 1.0
GO/CNF_{25}/TA_{10}	-89.7 ± 3.5	72.0 ±2.6

Table S4: Initial moisture content of the samples

Sample	Moisture Content (%)
CNF	8.3 ± 0.4
rGO/CNF_5	15.0 ± 0.8
rGO/CNF_{15}	12.0 ± 0.6
rGO/CNF_{25}	11.7 ± 0.6
rGO/CNF_{50}	11.6 ± 0.6
rGO/CNF_{25}/TA_5	11.7 ± 0.6
GO/CNF_{25}/TA_{10}	12.5 ± 0.6
